# Increased Memory Conversion of Naïve CD8 T Cells Activated during Late Phases of Acute Virus Infection Due to Decreased Cumulative Antigen Exposure

**DOI:** 10.1371/journal.pone.0014502

**Published:** 2011-01-06

**Authors:** Georgia Fousteri, Amy Dave, Amy Juedes, Therese Juntti, Bret Morin, Lisa Togher, Donna L. Farber, Matthias von Herrath

**Affiliations:** 1 Diabetes Center, La Jolla Institute for Allergy and Immunology, La Jolla, California, United States of America; 2 Columbia Center for Translational Immunology, Columbia University Medical Center, New York, New York, United States of America; BMSI-A*STAR, Singapore

## Abstract

**Background:**

Memory CD8 T cells form an essential part of protective immunity against viral infections. Antigenic load, costimulation, CD4-help, cytokines and chemokines fluctuate during the course of an antiviral immune response thus affecting CD8 T cell activation and memory conversion.

**Methodology/Principal Findings:**

In the present study, naïve TCR transgenic LCMV-specific P14 CD8 T cells engaged at a late stage during the acute antiviral LCMV response showed reduced expansion kinetics but greater memory conversion in the spleen. Such late activated cells displayed a memory precursor effector phenotype already at the peak of the systemic antiviral response, suggesting that the environment determined their fate during antigen encounter. In the spleen, the majority of late transferred cells exhibited a central memory phenotype compared to the effector memory displayed by the early transferred cells. Increasing the inflammatory response by exogenous administration of IFNγ, PolyI:C or CpG did not affect memory conversion in the late transferred group, suggesting that the diverging antigen load early versus later during acute infection had determined their fate. In agreement, reduction in the LCMV antigenic load after ribavirin treatment enhanced the contribution of early transferred cells to the long lasting memory pool.

**Conclusions/Significance:**

Our results show that naïve CD8 cells, exposed to reduced duration or concentration of antigen during viral infection convert into memory more efficiently, an observation that could have significant implications for vaccine design.

## Introduction

The generation of memory T cells is a crucial process for developing novel ways to prevent viral infections and certain forms of cancer [1]–[3]. Following exposure to antigen, T cells proceed through three defined phases: activation and clonal expansion, contraction and memory conversion [4]–[6]. Memory T cell development can be influenced by the antigen dose, the strength of the T cell receptor (TCR)-antigen interaction, costimulation, type of antigen presenting cells (APCs), the participation of CD4 helper/regulatory T cells and the cytokines and/or chemokine environment [7]–[13]. Two major memory T cell populations have been described based on their location and effector functions. Central memory (CM) T cells (CD44^hi^CD62L^hi^CCR7^+^) are located in secondary lymphoid tissues and possess little cytotoxic activity, while effector memory (EM) T cells (CD62L^lo^CCR7^−^), which reside in non-lymphoid tissues are cytotoxic and rapidly acquire effector function [14]–[18].

T cell activation and differentiation during the course of an infection can be influenced by changes in pathogen load [19]. As the amount of antigen decreases during the course of an acute infection, naïve T cells that are introduced at late stages seem to proliferate less and acquire different properties, such as decreased CD62L down-regulation [20], [21]. However, what determines EM versus CM and how the timing of viral infection affects this differentiation process are still open questions in the field. It is not known whether naïve T cells activated at the peak viral load during antigen abundance, versus peak viral clearance when the antigen load is low, have different capacities for T cell memory formation.

In addition to antigen levels, cytokines are known to play crucial roles in memory T cell survival and differentiation [22]. IL-7, one of the most well studied cytokines in mediating survival of naïve T cells seems to contribute to survival, and to a lesser extent, to basal homeostatic proliferation of memory T cells [23]–[25]. Upon TCR activation, IL-7Rα (CD127) is initially down-regulated on populations of activated effectors cells and increased CD127 levels was shown to determine effector CD8 T cells destined to become memory T cells [26]. More recent evidence suggests that coordinate expression of CD127 and killer cell lectin-like receptor G1 (KLRG-1), distinguishes short-lived effector cells (SLEC) from those destined to develop into long-lived memory T cells. SLEC display a KLRG-1^hi^CD127^lo^ phenotype, whereas memory precursor effector cells (MPECs) exhibit a KLRG-1^lo^CD127^hi^ phenotype [27]. The decision between SLEC and MPEC fates can be regulated by the inflammatory environment, which subsequently induces specific transcriptional programs in primed CD8 T cells [28]–[30]. In addition, the ability of effector CD8 T cells to produce IL-2 has been partially associated with stable memory development [31], [32]. Whether the inflammatory environment and/or antigen load are more predominant regulator of memory T cell development has not been resolved.

In the present study, we demonstrate that the timing at which naïve MHC class-I restricted, LCMV-specific, TCR transgenic (Tg) P14 T cell enter the primary immune response to LCMV can affect their expansion and capacity to differentiate into memory T cells. Naïve CD8 T cells activated in conditions of reduced antigen load during LCMV infection either through late introduction in infection or after ribavirin anti-viral treatment, converted into memory more efficiently than naïve CD8 T cells activated early during infection. As The majority of late transferred cells present at the peak of the response exhibited a KLRG1^lo^ phenotype, characteristic of memory precursor CD8 T cells [33]. In addition, late tranferred cells did not “contract” and remained as memory cells. They displayed a gradual shift from a CD44^hi^CD62L^lo^ (EM) phenotype to a CD44^hi^CD62L^hi^ (CM) phenotype and increased levels of IL-2 production, in agreement with previously published results [31], [32]. By contrast, naïve CD8 T cells transferred cells early in the course of LCMV infection, prior to peak viral load, were predominately EM, CD44^hi^CD62L^lo^. Increasing the inflammatory milieu after treatment with CpG, poly I:C or IFNγ had no significant effect on the late transferred cells, indicating that antigen load during infection was likely the main factor that determined their survival and memory conversion. In agreement, ribavirin treatment significantly reduced LCMV viral load and consequently the expansion and contraction phases of early transferred naive P14 TCR Tg cells. The conversion rate of early transferred naïve CD8 T cells into memory was significantly augmented, in ribavirin-treated versus untreated mice and was similar to that of late transferred cells. Our results suggest an inverse correlation between the degree of antigen-specific expansion and memory conversion for CD8 T cells, which may aid in the development of more effective vaccines and perhaps the treatment of autoimmune, CD8-mediated autoimmune diseases.

## Results

### Late recruitment of naïve CD8 cells during acute LCMV infection results in reduced expansion and contraction but increased memory conversion in the spleen

**Figure 1:**
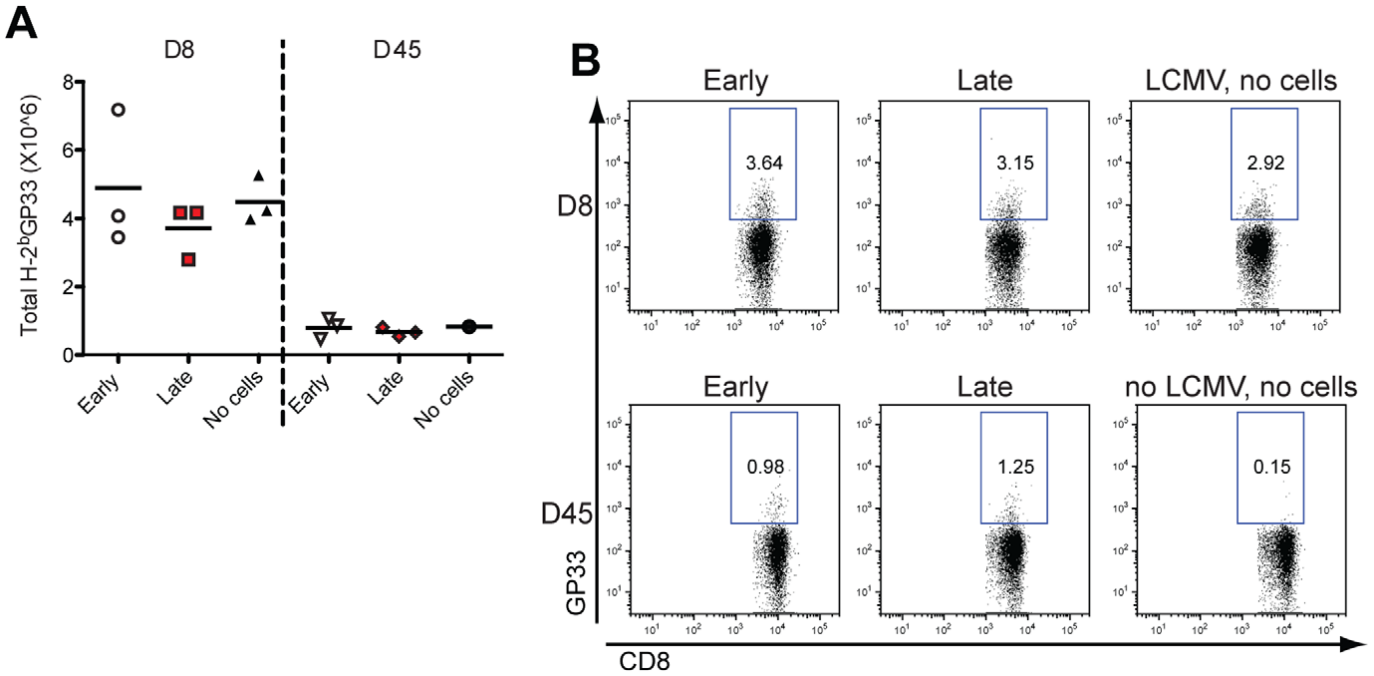
No significant effect in the endogenous GP33–41 LCMV-specific response after the transfer of 2×10^3^ P14 CD8 T cells. Spleens from mice receiving P14GFP^+^ on day 0 or 3 after infection were collected and analyzed by pentamer staining on day 8 and day 45 after infection (A). The total number of antigen-specific cells per spleen was calculated by multiplying the percent of GFP^−^CD8/GP33 pentamer double positive cells by the total number of cells isolated from the spleen of each mouse. A representative dot plot of percentage of GP33 specific CD8 T cells on day 8 and day 45 is shown in (B). Representative data are from one of two experiments. Differences are not statistically significant.

Introduction of small numbers of TCR-Tg, LCMV-specific CD8 cells accurately reproduces the natural anti-LCMV response without profoundly altering viral clearance and T cell expansion kinetics. In contrast, large number of naïve antigen-specific T cells can alter the physiological immune response and clearance of LCMV and the amount of the endogenous physiological cytokines and chemokines levels [34], [35]. Therefore, in order to better mimic the natural, acute CD8 anti-LCMV T cell response, we chose to adoptively transfer only relatively small numbers (2×10^3^) of traceable TCR-Tg LCMV-specific CD8 GFP^+^ T cells (GP_33–41_-specific –P14) into C57BL/6 LCMV Arm infected mice. To study how the timing at which a naïve T-cell enters an antiviral response affects its proliferation and memory conversion, P14/GFP^+^ CD8 T cells were either transferred on day 0 (early) or day 3 (late) postinfection. We reasoned that cells that were transferred later post infection would have less opportunity to encounter viral antigen in vivo, because LCMV antigenic load usually peaks 2–3 days after infection and virus is cleared by day 7 from most organs it shows tropism [36], [37]. To circumvent differences in immune and viral kinetics between the day 0 and day 3 groups, mice that received P14/GFP^+^ cells on day 0 also received P14/GFP^−^ cells on day 3, while mice that received P14/GFP^+^ cells on day 3 had also received P14/GFP^−^ cells on day 0 (Table 1). As shown in Fig. 1, numbers of GFP^−^ GP_33–41_-specific effector cells on day 8 and day 45 p.i. in both groups were identical and not significantly different from mice that had received no P14 cells. Thus, introduction of low numbers of P14 T cells did not significantly alter the general kinetics of the antiviral immune response and therefore is a valid approach to differentially track early and late transferred cells within the GP_33–41_-specific T cell response against acute LCMV infection.

**Table 1:** Late and early transferred group of P14 LCMV-specific naïve CD8 T cells.

Group	D = 0 cells transferred	D = 3 cells transferred
Early	2×10∧3 P14/GFP^+^	2×10∧3 P14/GFP^−^
Late	2×10∧3 P14/GFP^−^	2×10∧3 P14/GFP^+^

**Figure 2:**
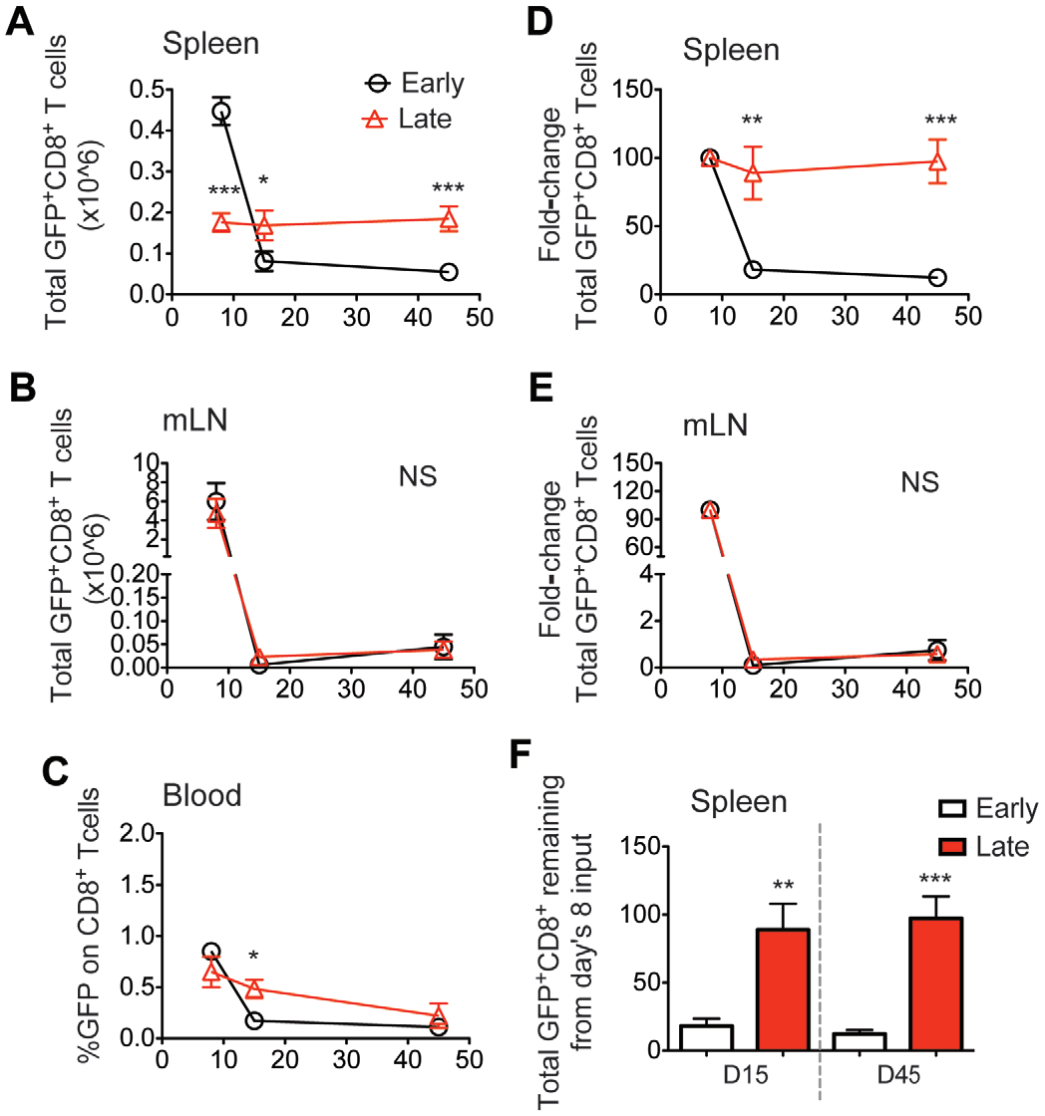
Late recruited naïve CD8 cells convert at a higher efficiency to memory cells in the spleen. Spleen, blood and mesenteric lymph node (mLN) cells from mice receiving P14/GFP^+^ early (day 0) or late (day 3) post infection (and GFP^−^ cells on days 3 and 0, respectively) were analyzed on days 8, 15 and 45 p.i. with flow cytometry. The total CD8^+^GFP^+^ number or the GFP^+^ percentage gated on CD8 T cells is depicted for each tissue (A–C). The CD8^+^GFP^+^ percentage for the spleen and mLN was multiplied with its respective total lymphocyte number. The fold-change from day 8 in the total GFP^+^ is shown in (D–E). The fold-change was calculated as follows: the ratio of the measured CD8^+^GFP^+^ percentage or the total number on day 8, minus day 15 or day 45, divided by the value on day 8, times 100. (i.e. CD8^+^GFP^+^ % for day 8 = 1 and day 15 = 0.5, while day 45 = 0.2, fold-change on day 15 = [(1−0.5)/1]x100 = 50% and on day 45 = [(1−0.2)/1]x100 = 20%). The total GFP^+^ in CD8 T cells output on day 15 and day 45 from day 8's input is represented (F). *, p<0.05, **, p<0.005, ***, p<0.001, NS, not statistically significant. Similar data were obtained from at least five independent experiments with three to four mice per group.

C57BL/6 mice were analyzed on days 8, 15 and 45 p.i. for the presence of GFP^+^ cells. The percentage or total numbers of transferred P14/GFP^+^ CD8 T cells in the spleen, blood, and mesenteric lymph nodes (mLN) for early and late transferred cells are shown in Fig. 2*A*–*C*. As expected, the response kinetics of the P14 T cells transferred early were similar to the endogenous antigen-specific populations: defined clonal expansion, contraction and memory conversion phases were observed. In contrast, P14/GFP^+^ cells transferred 3 days late displayed only a small degree of proliferation particularly and consistently in the spleen, showing that T cells that encounter their cognate antigen early during the immune response make up the majority of the responding population. The frequency of memory P14/GFP^+^ cells within the CD8 population was identical between early and late transferred groups in all lymphoid organs analyzed (data not shown). However, when we determined the fate of the total P14 transferred cells present at the peak of the response by analyzing the percentage of cells remaining in the contraction and memory phases (normalized for D8, fold-change), a much greater output in memory cells in the late transferred compared to the early transferred cells was seen in the spleen (Fig. 2*D*–*F*). Overall, P14 T cells introduced early during the initial phase of the antiviral immune response exhibited much greater expansion and contraction rates compared to late ones in the spleen. On the other hand, cells exposed to reduced viral antigen in vivo (late), do not expand to the same extent, yet convert to memory T cells with greater efficiency.

### The majority of late transferred CD8 T cells present at the peak of the anti-LCMV response convert to memory displaying a CM phenotype

**Figure 3:**
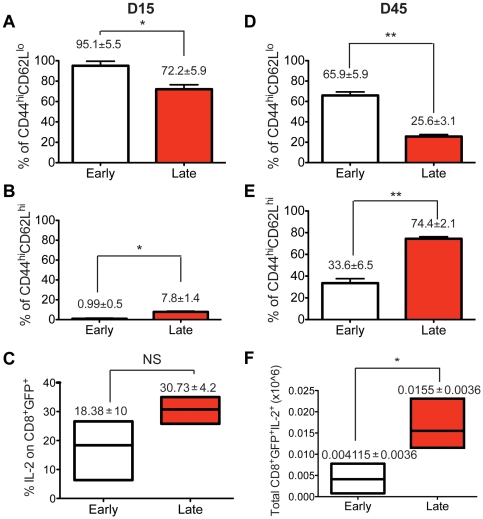
Late transferred CD8 T cells display a gradual shift from a CD62L^lo^ (EM) phenotype to a CD62L^hi^ (CM) phenotype. C57BL/6 mice infected with LCMV received purified CD8 T cells isolated from naïve P14/GFP^+^ mice on day 0 and day 3. On days 15 and 45, GFP^+^CD8 T cells from the spleen, blood and mLN were examined for the expression of CD44 and CD62L. The percent expression of CD44^hi^ and CD62L^lo^ on CD8^+^GFP^+^ gated cells is shown in (A) and in (D) for day 15 and 45 respectively. The percentage of CD44^hi^/CD62L^hi^ expression gated on CD8^+^GFP^+^ is depicted in (B) and (E) for day 15 and 45 respectively. The percentage and total number of CD8^+^GFP^+^ cells producing IL-2 on day 45 are displayed accordingly in (C) and (F). *, p<0.05, **, p<0.005, NS, not statistically significant. Similar data were obtained from at least two independent experiments.

It is evident from the results discussed above that the majority of naïve T cells recruited late during the immune response displayed a higher degree of memory conversion in the spleen. Our results suggest that the cells recruited later during the acute anti-LCMV response can become memory cells at higher efficiency than naïve CD8 cells that join the antiviral immune response early and expand maximally. Next, we wanted to investigate whether the relative contribution to the CM or EM CD8 T cell pool differs between the early and late transferred cells. In order to compare memory subset development between the early and late transferred cells, additional characterization based on CD44 and CD62L expression was done. We chose CD62L since it is a key marker that distinguishes EM from CM memory T cell subsets along with the expression of CD44. Of note, all P14 cells transferred late or early displayed CD44 upregulation at the peak of the response in the spleen (data not shown). Later on, at the contraction phase, more than 70% of the cells displayed an EM phenotype CD44^hi^CD62L^lo^ in both groups (Fig. 3*A*–*B*). Interestingly though, while in the early transferred group almost one third of the cells displayed CM phenotype at the memory phase, a much greater proportion of cells (>70%) in the late transferred group displayed CM features (Fig. 3*D*–*E*). These phenotypic characteristics were somewhat different in the mLN and blood, since in both early and late transferred cells, preferential high levels of both CD44 and CD62L expression were seen at the contraction and memory phase, indicating that these cells fall within the CM population (data not shown).

In addition, in order to differentiate between the functional capacities of CM and EM cells, IL-2 production from the CD8^+^GFP^+^ population was measured 45 days after infection, following ex-vivo stimulation with the class I, P14-specific peptide GP_33–41_ (Fig. 3*C&F*). Indeed, IL-2 production was greatly enhanced in terms of total number by late transferred cells, consistent with their predominant CM phenotype. This preferential IL-2 production together with re-expression of CD62L by the late transferred group suggests that the strength of antigen stimulation received during the priming phase of the response was reduced compared to the early transferred cells [*20*], [*21*]. In addition, memory cells generated from these late transferred CD8 T cells adopt homing properties, characteristic of the CM subset.

### Early and late transferred CD8 cells display similar functional characteristics at the effector and memory phases

**Figure 4:**
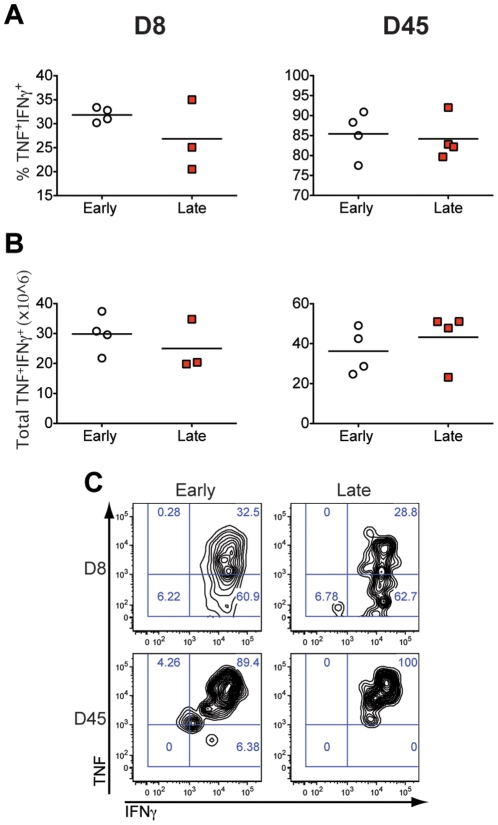
Early and late recruited CD8 cells display similar functional characteristics during the antiviral effector and memory phases. Spleens were collected and analyzed 8 and 45 days after infection. Splenocytes were cultured with the LCMV MHC-I peptide GP33 before staining for intracellular IFNγ and TNF. The percentage (A) and total number (B) of TNF/IFNγ double positive cells after gating on the CD8^+^GFP^+^ population are shown. The total number of antigen-specific cells per spleen was calculated by multiplying the percent of TNF/IFNγ double positive cells by the total number of cells isolated from the spleen of each mouse. A representative dot plot of TNF and IFNγ expression by GFP^+^CD8 gated cells is shown in (C). Representative data are from one of two experiments. Differences are not statistically significant.

As effector T cells differentiate into memory cells, they acquire a CM or EM phenotype and retain the potential to rapidly produce IFNγ and TNF when exposed to antigen. We compared the ability of P14 early and late transferred cells to secrete cytokines in response to antigen during the primary antiviral effector and memory phase. As shown in Fig. 4, intracellular cytokine staining after gating on P14/GFP^+^ cells following stimulation with the class I-restricted epitope GP_33–41_ showed no difference in IFNγ and TNF production at the peak of the response day 8 p.i. (D8) between early and late transferred groups. Similar analysis conducted at the memory stage day 45 p.i. (D45), in which early transferred cells displayed a predominantly EM phenotype while late transferred cells had become CM in the spleen, showed that both groups exhibited similar cytokine production characteristics indicative of functional memory. In conclusion, although cells engaged late in the immune response are exposed to less inflammatory signals and antigen and therefore proliferate and contract less, they acquire normal CM characteristics and are fully able to produce antiviral effector cytokines.

### Late transferred cells display a memory precursor phenotype at the peak of the response

**Figure 5:**
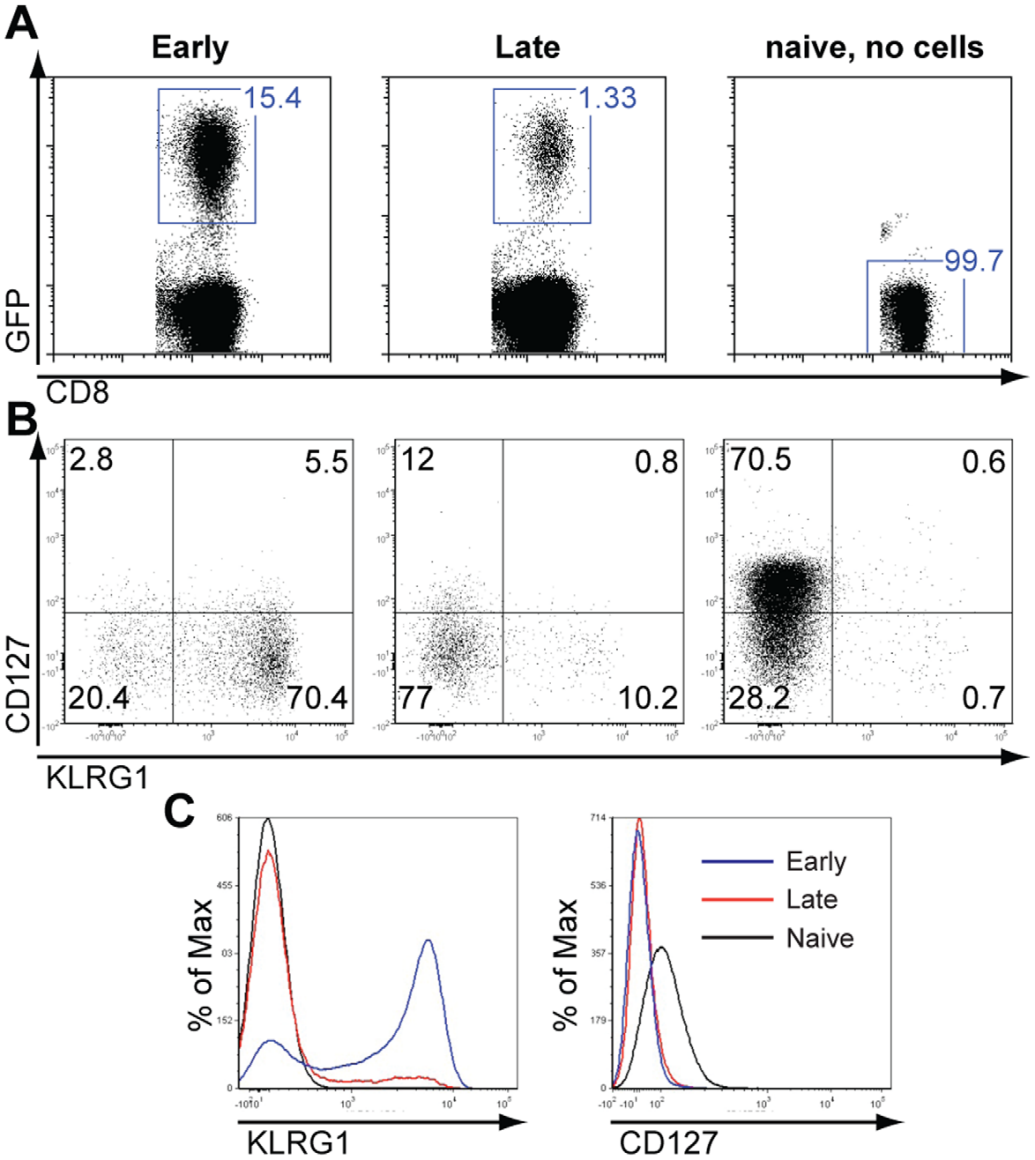
Majority of late transferred cells display a memory precursor phenotype at the peak of the anti-LCMV response. Naïve P14 GFP^+^ cells from the late and early transferred groups were analyzed by flow cytometry for the expression levels of CD127 and KLRG1. The GFP^+^ cell percentage within the total CD8 population is depicted in the first 3 horizontal graphs for the early and late transferred cells and the naïve control (A). A representative dot plot of CD127 and KLRG1 expression is shown for all three groups (B). Histogram overlay for the expression of KLRG1 and CD127 in P14/GFP^+^CD8^+^ early versus late transferred cells and naïve controls at the peak of the response (C).

Recently it has been suggested that memory cell precursors can already be identified at the peak of the response by high levels of CD127 in conjunction with low levels of KLRG1 expression. Since we observed that the majority of naïve P14 cells that enter the immune response at a later time point remain as memory, we performed a phenotypic analysis in order to examine whether the late transferred cells display a memory precursor phenotype early, by the eighth day after infection with LCMV. To this end, naïve P14/GFP^+^ cells from late and early transferred groups were analyzed by flow cytometry for the expression levels of CD127 and KLRG1. Interestingly, the majority of P14 transferred cells had downregulated the expression levels of CD127 (Fig. 5), which was even more pronounced in the early transferred group. Importantly, comparison of KLRG1 levels detected within the P14/GFP^+^ cells at the peak of the response (Fig. 5*A*) between early and late transferred cells showed a strong correlation of greater memory formation in the later group with the lower expression of KLRG1 levels in these samples (Fig. 5*B*–*C*). Based on the recent classification for memory precursor effector cells (MPECs), the IL-7Rα^hi^/KLRG1^lo^ cell frequency was much greater in the late (13.8%) than early (2.8%) transferred P14/GFP^+^ cells at the peak of the anti-LCMV response (Fig. 5*B*). Collectively, these data indicate that naïve CD8 T cells recruited later in the antiviral immune response largely convert into memory and their fate is determined as early as the initial stages of their activation.

### Duration of antigen exposure is the decisive factor for memory T cell fate

Our results prompted us to investigate the signals that are necessary to enhance memory conversion of the late transferred cells. Initially we hypothesized that their greater memory conversion was either due to differences in i) antigen load (less cumulative antigen exposure over time ii) inflammatory stimuli/activation status of the APCs or iii) due to greater IL-7Rα levels expressed by P14 cells entering the response at a later stage. In order to identify parameters other than viral antigenic load that differ and could affect memory conversion of antiviral CD8 T cells, mice that had received naïve P14/GFP^+^ cells late were treated the day after the cell transfer with polyI:C (100 µg/mouse), CpG (200 µg/mouse) or recombinant IFNγ (50 ng/mouse). As shown in Fig. S1, treatment with any of these three regiments did not alter memory conversion. In parallel to these experiments, we were able to recapitulate the enhanced memory conversion of P14 early transferred naïve CD8 T cells after recombinant IL-7 treatment as previously described (30) (data not shown).

**Figure 6:**
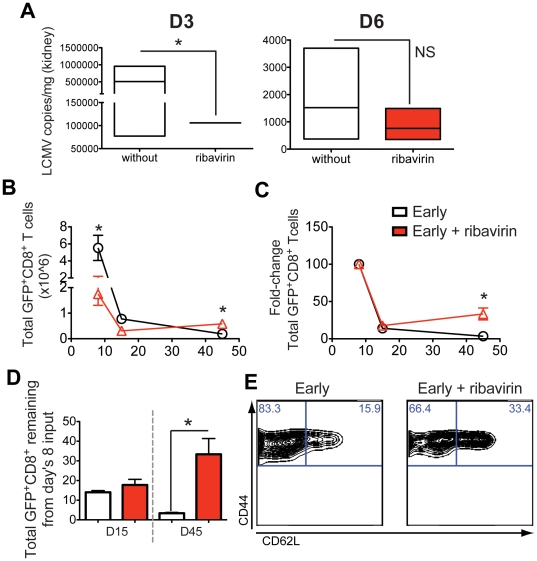
Ribavirin treatment reduces LCMV antigenic load impacting memory CD8^+^ T cell development. Mice were treated with ribavirin (Rebetol) orally on a daily basis. Each mouse received 8 mg ribavirin for 10 consecutive days, starting seven days prior to LCMV Arm infection and continuing until three days after infection. Mice received P14/GFP^+^ CD8 T cells the same day of infection. Viral load was quantified with qPCR in the kidneys of the infected mice 3 and 6 (A) days after infection. Significant decrease in viral load was detected only on day 3, *, p<0.05, NS, not statistically significant. The total CD8^+^GFP^+^ number (B) and Fold-change (C) was calculated as in Fig. 2 for the spleen for days 8, 15 and 45 after infection. The percentage of total CD8^+^GFP^+^ cells remaining on days 15 and 45 from day's 8 input is represented graphically in (D). Representative flow cytometry plots showing the CD44/CD62L profile of the cells on day 45 after infection gated on CD8^+^/GFP^+^ is shown in (E). Almost double CM (CD44^hi^/CD62L^hi^) cell frequency can be detected in the group of mice treated with ribavirin.

In order to address in more detail whether cumulative antigen exposure over time was the decisive factor for T cell fate determination, we took advantage of an additional approach. P14/GFP^+^ cells were transferred to LCMV infected recipients early, which were previously and continuously treated with ribavirin (Rebetol) orally on a daily basis. Ribavirin is a nucleoside analog that is an effective antiviral treatment against arenaviruses [38]. Each recipient mouse received 8 mg ribavirin for 10 consecutive days, starting seven days prior to LCMV infection and continuing until three days after infection. As shown in Fig. 6*A*, ribavirin treatment reduced the LCMV copies in the kidney significantly three days but not six days after infection. The reduction in virus levels caused the endogenous GFP^−^/GP33-pentamer^+^ and transferred GFP^+^/P14 cells to drop at the peak of the response (Fig. 6*B* and data not shown). However, and in agreement with our late versus early transfer approach, memory development was favored (Fig. 6*B*–*D*). Although in this scenario the contraction phase was not diminished compared to late transfer experiments, cells contributed again more effectively to the ensuing memory pool. Importantly, at the memory phase, P14 early transferred cells in the group of mice treated with ribavirin, there was an increased proportion of CD62L^hi^ CM cells (Fig. 6*E*). Altogether, our results suggest that by reducing the antigen load alone, we do not compromise memory T cell development but rather promote differentiation of the CM subset.

## Discussion

**Figure 7:**
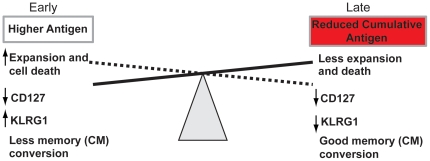
Antigenic load affects the expansion and contraction phase of an anti-LCMV response positively whereas it is inversely related to memory conversion. This schematic represents that when P14/GFP^+^ T cells were introduced at a later time point of acute LCMV response a much less expansion followed by almost no contraction was observed. Nevertheless, a much larger proportion of those cells found on D8 converted and contributed to the memory cell pool.

In this study we demonstrated that LCMV-specific naïve CD8 T cells that experience reduced cumulative antigen exposure during the anti-LCMV response convert more efficiently into memory CD8 T cells. The majority of cells activated late exhibit an activated phenotype with CD44 upregulation and CD62L downregulation at the acute and contraction phases indicative of previous antigen encounter (data not shown and Fig. 3*A*–*B*). However, cells exposed to low levels of antigen become imprinted with a distinct long-term differentiation program: such cells do not expand and consequently do not contract to the same extent, while they primarily survive as CM cells (Fig. 7). Most CD8 T cells recruited at a later stage downregulate IL-7Rα levels, while maintaining low levels of KLRG1 expression at the peak of the response (Fig. 5). Given their high conversion rate into memory, perhaps a significant proportion of KLRG1^lo^/IL-7Rα^lo^ cells should be considered as memory precursor cells, which contradicts the current classification of MPECs as IL-7Rα^hi^ cells. In earlier studies, constitutive IL-7Rα expression had a minimal effect on the formation and function of effector and memory CD8 cells, suggesting that IL-7Rα levels do not identify memory CD8 T cell precursors (31). KLRG1^lo^ cells though, irrespective their IL-7Rα levels, seem to give rise to IL-7Ra^hi^ long-lived memory cells (33). Taken together, KLRG1 and to a lesser extent IL-7Rα levels seem to best define memory precursor frequency as early as at the peak of the response.

Since naïve cells entering the immune response at a later time point express higher CD127 levels, we investigated whether blocking IL-7Rα during the contraction phase would affect the outcome of memory T cell development. However, we did not observe consistent effects after CD127 blockade on memory CD8 conversion (data not shown). Together, our results suggest that anti-CD127 treatment at the contraction phase does not have a clear impact on naïve CD8 T cell memory formation, similar to what was previously described [12].

Exogenous treatment with inflammatory stimuli could not reverse preferential memory conversion of the late recruited cells, suggesting that the antigenic load is likely the main factor that contributes to memory formation, possibly determined by the number of T cell to APC contacts. Cells that encounter more antigen or more recently infected APCs receive stronger antigenic stimulation and co-stimulation, proliferate more vigorously and therefore experience greater activation-induced cell death or become imprinted as senescent effectors or SLECs, thus making conversion to memory less likely. In agreement, treatment with ribavirin prior to early P14/GFP^+^ cell transfer, while reducing the viral load and the expansion of cells at the peak of the response, had a positive impact on memory formation. Overall, our results support the decreasing-potential model (6), which proposes that one of the main factors controlling memory output of a given population recruited in the immune response is the duration of antigen exposure during priming. CD8 T cells recruited later in the response, despite receiving a weak stimulation, become imprinted with a memory differentiation program, perhaps because the received signal is not adequate enough to trigger the death pathway. In addition, full differentiation into effector cells is not prerequisite for memory conversion [39]. In agreement with our findings, during persistent viral infection, prolonged and strong antigenic encounter decreases the contribution to the memory pool by constantly eliminating effector T cells and MPECs [4], [40].

Recent reports have also examined the effect of timing and antigen load on T cell priming and memory development [21], [34], [35]. In one study using a vaccinia viral infection model, the initial burst size of hemagglutinin (HA)-specific CD8 effector T cells in response to recombinant vaccinia virus encoding HA (rVV-HA) correlated with the magnitude of the long-term memory pool size (37). The major caveat of this study was that rather large numbers of cells were transferred (1×10∧5) without the inclusion of control co-transfers, which may have masked the endogenous physiological response. Another study, using a recombinant vesicular stomatitis virus (VSV) -expressing OVA (VSV-OVA) infection model, found that “latecomer” OT-I cells were not preferentially recruited to the surviving memory pool (21), contrasting our results where naïve CD8 T cells activated later in infection efficiently converted to memory T cells. However, in the VSV study, engrafted OT-I cells exceeded the natural OVA-specific precursor frequency, with potential effects on the physiological response and memory formation. Here, we have recapitulated the endogenous physiological anti-LCMV response by transfer of low numbers of virus-specific CD8 T cells with minimal impact on the endogenous anti-viral CD8 T cell response. With this system, we found that specific low frequency naïve CD8 T cells acquire distinct differentiation fates depending on the antigen dose and to a lesser extent the inflammatory response. There have been recent examples of residual antigen persisting long after infection in mouse virus infection models [40], [41]. Our results indicate that memory formation may continue to occur in conditions of low antigen load even after virus is effectively cleared.

Studies using a novel barcode technology to mark individual T cells showed that single naïve CD8 T cells could yield heterogeneous populations of effector and memory CD8 T cells (38). These results suggest that effector and memory fates are not imprinted by distinct APC or antigen/timing signals delivered during initial priming. We also found that naïve CD8 T cells can adopt multiple fates under a variety of conditions and that the timing of activation during infection is an important factor. In summary, our results support that sufficient and effective memory conversion could be achieved in vivo even at a reduced antigen dose, providing important implications for vaccine design.

## Materials and Methods

### Mice

Eight- to 10-wk-old male C57BL/6 mice were purchased from Jackson laboratories. Naïve TCR Tg P14 mice were bred to GFP^+^ (both on the C57BL/6 background) to obtain GP33-specific, GFP^+^ double Tg mice (P14/GFP^+^). Expression of both transgenes was confirmed by flow cytometry after testing for GFP, Vα2, and Vβ8.1/8.2 expression. All mice were maintained under specific-pathogen-free conditions at the La Jolla Institute for Allergy and Immunology (LIAI) and handled in accordance with the LIAI Animal Care and Use Committee approved protocols. The experiments for this study were conducted according the approved mouse protocol: #AP117-MvH2-0510 [600] (approved 05/25/10) “Viruses and autoimmunity”.

### CD8 T cell negative selection and adoptive transfer

Naïve CD8 T cells were purified from splenocytes of P14/GFP^+^ or P14/GFP^−^ mice by negative selection using the following purified monoclonal antibodies: anti-B220, anti-CD4, anti-CD11c, anti-FcγRII (clone 2.4G2), anti-mouse MHC Class II I-A/I-E and anti-CD11b. All antibodies were from BioLegend (San Diego, CA, USA). CD8 T cells were then purified by magnetic separation using the Sheep anti-Rat IgG coated Dynabeads (Invitrogen, San Diego, CA, USA). Before transfer, cells were washed extensively with HBSS-HEPES buffer. 2×10^3^ of naïve P14/GFP^+^ or P14/GFP^−^ CD8 T cells were adoptively transferred into the tail vein (intravenously, i.v.) on days 0 and 3 post infection (p.i.). Mice that received P14/GFP^+^ cells early, received P14/GFP^−^ late, whereas the ones that received P14/GFP^+^ cells late, received P14/GFP^−^ early.

### Virus

Mice were infected with 10^4^ PFU of LCMV strain Armstrong (53b) by intraperitoneal (i.p.) injection.

### Treatments

Mice were treated once on day 4 after infection with LCMV with 100 µg/mouse polyI:C (Amersham Pharmacia) i.p. or 50 ng/mouse recombinant mouse IFNγ (BD Pharmingen, San Diego, CA, USA) i.p. or with 200 µg/mouse CpG i.v. Anti-CD127 (125 µg/mouse) treatments (clone SB/14 BD Pharmingen or clone A7R34 Biolegend) were conducted i.p. in the contraction phase on days 8-10-12-14 after LCMV infection. Mice were treated with ribavirin (Rebetol, USP- NDC 0085-1318-01) orally on a daily basis. Each mouse received 8 mg ribavirin for 10 consecutive days, starting seven days prior to LCMV infection and continuing until three days after infection.

### Flow cytometry

Single cell suspensions were prepared from spleen, peripheral blood and mesenteric lymph nodes (MLN) from all groups. After a 2.4G2 block step, cells were stained with the conjugated antibodies for cell surface markers. PE-conjugated H2-D^b^/GP33 pentamers were purchased from ProImmune and stained as previously described [42]. Directly conjugated antibodies, CD8-PerCP, CD62L-APC, CD127-PE or PeCy7 (BD Pharmingen), CD44-APCCy7, CCR7-PeCy7, KLRG1-PE (e-Bioscience, San Diego, CA, USA) and CD25-PB (Biolegend) were used. For surface staining, cell suspensions were incubated at 4°C for 30 min. After surface staining, cells were fixed in 4% paraformaldehyde (Sigma-Aldrich). D^b^GP33 and class I pentamers were obtained as PE conjugates from Proimmune and used as described previously. For intracellular cytokine analysis, single cell suspensions were stimulated in vitro for 3 hours with 1 µg/ml MHC class I-restricted viral peptides GP_33–41_ (GP33) (Abgent, San Diego, CA, USA). Cells were stained for surface expression of CD4 and CD8, fixed, permeabilized, and stained for intracellular IL-2, IFNγ and TNF. After staining, cells were processed on LSRII (BD Biosciences) and results were analyzed using FlowJo (Tree Star).

### Quantitative PCR

Kidney samples were surgically removed and frozen at −80°C, then weighed and homogenized. RNA was isolated using the RNAqueous mini spin column based system (Ambion). RNA was eluted from RNAaqueous spin columns in a volume of 20 µl. 8.5 ul of RNA was used in a 10 µl cDNA reaction with SuperScript III Reverse Transcriptase (SSIII) (Invitrogen, Carlsbad, CA) and a GP-R primer (S pos. 970–991), GCAACTGCTGTGTTCCCGAAAC GP-R at 55°C for 1 hr in a programmed PCR thermocycler. 10 µl of cDNA was used as template for a 25 µl qPCR reaction using SYBR Green kit (Roche), plated in 96 well plate format and run on a LightCycler 480 (Roche). Amplification was done for 40 cycles, with each cycle consisting of two steps: 95°C, 15 sec; 60°C, 30 sec. All qPCR samples ran in triplicate, with water as a negative control and LCMV as a positive control. Standard curves were generated using linearized pSG5-GP plasmid.

### Statistical analysis

Data are expressed as a mean ± SD. The statistical significance of the difference between means was determined using the two-tailed Student's *t*-test. *, p<0.05, **, p<0.005, ***, p<0.001.

## Supporting Information

Figure S1 Recruitment of late transferred cells into the memory T cell pool is not influenced by inflammatory agents. Mice were infected with acute LCMV and received P14/GFP^+^ CD8 T cells the same day or 3 days after infection. Groups of mice were treated 1 day after cell transfer with recombinant mouse IFNγ, polyI:C, CpG, or no treatment, as described in the Materials and Methods section. The percentage of GFP^+^ cells remaining on day 28 from day 8's input is represented graphically.
(1.01 MB TIF)
