# Supplementary figures and images for: Increased Memory Conversion of Naïve CD8 T Cells Activated during Late Phases of Acute Virus Infection Due to Decreased Cumulative Antigen Exposure
Source: PLoS One. 2011 Jan 6;6(1):e14502. doi: 10.1371/journal.pone.0014502 (PMC3017078; doi:10.1371/journal.pone.0014502)

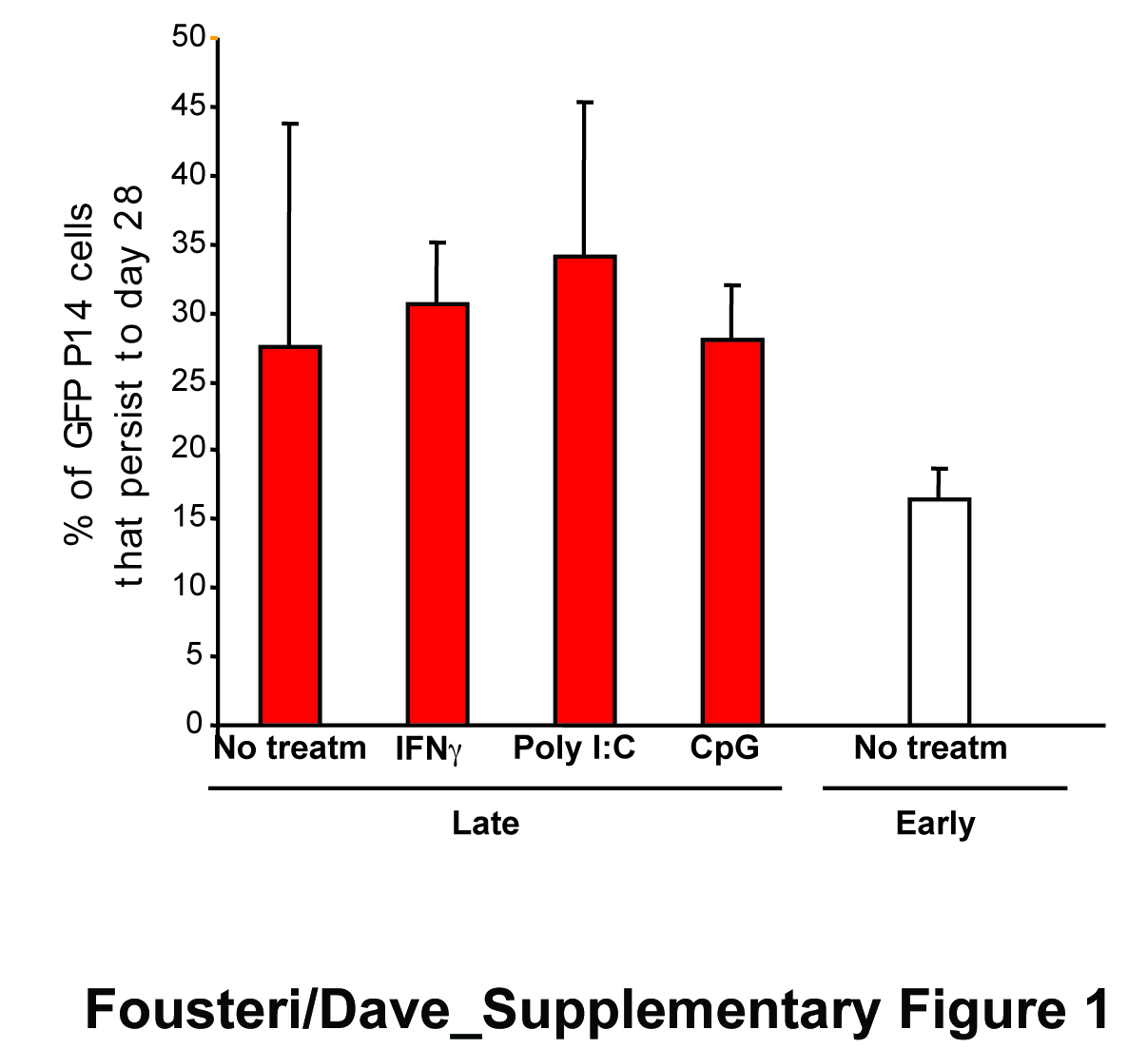

Supplement: Figure S1 — Recruitment of late transferred cells into the memory T cell pool is not influenced by inflammatory agents. Mice were infected with acute LCMV and received P14/GFP+ CD8 T cells the same day or 3 days after infection. Groups of mice were treated 1 day after cell transfer with recombinant mouse IFNγ, polyI:C, CpG, or no treatment, as described in the Materials and Methods section. The percentage of GFP+ cells remaining on day 28 from day 8's input is represented graphically. (1.01 MB TIF) [file pone.0014502.s001.tif]
